# Plasma Levels of Aminothiols, Nitrite, Nitrate, and Malondialdehyde in Myelodysplastic Syndromes in the Context of Clinical Outcomes and as a Consequence of Iron Overload

**DOI:** 10.1155/2014/416028

**Published:** 2014-01-14

**Authors:** Kristýna Pimková, Leona Chrastinová, Jiří Suttnar, Jana Štikarová, Roman Kotlín, Jaroslav Čermák, Jan Evangelista Dyr

**Affiliations:** ^1^Department of Biochemistry, Institute of Hematology and Blood Transfusion, U Nemocnice 1, 128 00 Prague 2, Czech Republic; ^2^Clinical Department, Institute of Hematology and Blood Transfusion, U Nemocnice 1, 128 00 Prague 2, Czech Republic

## Abstract

The role of oxidative stress in the initiation and progression of myelodysplastic syndromes (MDS) as a consequence of iron overload remains unclear. In this study we have simultaneously quantified plasma low-molecular-weight aminothiols, malondialdehyde, nitrite, and nitrate and have studied their correlation with serum iron/ferritin levels, patient treatment (chelation therapy), and clinical outcomes. We found significantly elevated plasma levels of total, oxidized, and reduced forms of cysteine (*P* < 0.001) , homocysteine (*P* < 0.001), and cysteinylglycine (*P* < 0.006) and significantly depressed levels of total and oxidized forms of glutathione (*P* < 0.03) and nitrite (*P* < 0.001) in MDS patients compared to healthy donors. Moreover, total (*P* < 0.032) and oxidized cysteinylglycine (*P* = 0.029) and nitrite (*P* = 0.021) differed significantly between the analyzed MDS subgroups with different clinical classifications. Malondialdehyde levels in plasma correlated moderately with both serum ferritin levels (*r* = 0.78, *P* = 0.001) and serum free iron levels (*r* = 0.60, *P* = 0.001) and were significantly higher in patients with iron overload. The other analyzed compounds lacked correlation with iron overload (represented by serum iron/ferritin levels). For the first time our results have revealed significant differences in the concentrations of plasma aminothiols in MDS patients, when compared to healthy donors. We found no correlation of these parameters with iron overload and suggest the role of oxidative stress in the development of MDS disease.

## 1. Introduction

Myelodysplastic syndromes (MDS) are a heterogeneous group of clonal hematological disorders, characterized by ineffective hematopoiesis and a high risk of transformation into acute myeloid leukemia (AML). Although the origin of MDS development is not fully understood, it has been determined that oxidative stress plays an important role in the initialization and disease progression of MDS [1].

One of the suggested mechanisms causing oxidative stress in MDS is attributed to a non-transferrin-bound iron (NTBI or free iron), which has been found in higher levels in the early stages of MDS patients receiving frequent red blood cell (RBC) transfusions [2]. Several studies have found elevated levels of oxidative stress markers (reactive oxygen species) and reduced levels of antioxidants (reduced glutathione (GSH)) in MDS patients and their correlation with serum ferritin levels [3, 4]. However, increased oxidative stress was revealed, even in the patients not receiving transfusions [5]. The presence of several other oxidative stress markers has been described in patients with established MDS, independent of iron or ferritin levels [6–8].

Oxidative stress, the imbalance in prooxidative and antioxidative processes, in favour of the first, acts through reactive oxygen species (ROS) and reactive nitrogen species (RNS). Oxidative status is reflected in blood plasma by actors of oxidative stress (free radicals and their metabolites), their products such as modified biomacromolecules, products of lipid peroxidation (malondialdehyde (MDA), 4-hydroxynonenal), and changes in the concentrations of compounds involved in antioxidant defense (enzymes, macromolecular and low-molecular-weight antioxidants, e.g., aminothiols).

Oxidative stress has been related to the origin and progression of a growing number of human diseases; however, their clear correlation is far from being proven [9]. Key factors influencing the evaluation of oxidative stress and its relation to the disease pathogenesis have been pointed out. They are (1) the choice of biomarker(s) and/or the biological system(s) for the analyses; (2) pitfalls in preanalytical and analytical methods for assessing oxidative stress; and (3) scientific misconduct [9]. Considering discussed factors simultaneous determination of plasma oxidative stress actors, their products, and antioxidant defense molecules is necessary to investigate the role of oxidative stress in the pathogenesis of MDS. The only work simultaneously evaluating oxidative stress markers and antioxidant defense molecules was done by Ghoti et al. in blood cells; however, to the best of our knowledge we have not found any work evaluating oxidative stress markers and antioxidant defense molecules in plasma of MDS patients and their relationships with each other and with iron and ferritin levels.

The aim of this study has been to assess the oxidative status of MDS patients and healthy donors by the evaluation of levels of antioxidant defense molecules (plasma total, oxidized, and reduced forms of aminothiols: GSH, cysteine (Cys), cysteinylglycine (CG), and homocysteine (Hcys)), marker of oxidative stress (MDA), and metabolites of NO (nitrite (NO_2_^−^) and nitrate (NO_3_^−^)) Although plasma nitrite and nitrate are not significant biomarkers of oxidative stress, they reflect NO species in plasma. We further estimated their relationship with serum iron/ferritin levels and clinical outcomes in MDS patients.

## 2. Materials and Methods

### 2.1. Materials and Reagents

All chemicals were obtained from Sigma-Aldrich (St. Louis, MO, USA) unless otherwise specified. All reagents employed were of analytical grade or higher purity, and all aqueous solutions were prepared using HPLC-grade water.

### 2.2. Blood Plasma Samples

Blood samples were retrospectively collected from 61 patients with MDS, diagnosed at the Institute of Hematology and Blood Transfusion, Prague, Czech Republic, and from 23 healthy volunteers. None of the patients had received any specific therapeutic agents prior to the study. Patients were not on any special diet prior to the study. All individuals tested agreed to the study at the time of blood collection. All samples were obtained in accordance with the Ethical Committee regulations of the Institute of Hematology and Blood Transfusion, Prague; and with a release of informed consent. Blood samples were drawn from patients and controls in a vacutainer tube containing EDTA for plasma, or containing beads coated with a clotting activator for serum (serum iron and serum ferritin determination); the tubes were immediately cooled on an ice bag and centrifuged as soon as possible at 4000 ×*g*, for 5 min, at 4°C. Serum and plasma samples were stored in the dark at −70°C until the analysis.

Diagnoses were made according to the 2008 WHO and FAB classification systems. Patients with unclassified MDS, Fanconi anemia, chronic myeloid leukemia, autoimmune thrombocytopenia, and congenital anemia have been excluded from the analysis. Thus, the following categories were considered: refractory anemia (RA), MDS with isolated del(5q) (RA-5q), refractory anemia with ringed sideroblasts (RARS), refractory cytopenia with multilineage dysplasia (RCMD), RCMD with ringed sideroblasts (RCMD-RS), RCMD with 5q deletion (RCMD-5q), RCMD with reactive monocytes, RA with an excess of blasts-1 (RAEB-1), RA with an excess of blasts-2 (RAEB-2), RA with an excess of blasts in transformation (RAEB-T), myelodysplastic/myeloproliferative syndromes (MDS/MPS), MDS/MPS with 5q deletion, acute myeloid leukemia (AML M2), and MDS-RAEB1 plus SC-non-Hodgkin lymphoma. In our study there were 19 patients with cardiovascular event and 28 patients with hypertension.

All the healthy subjects enrolled in this study were asymptomatic and none of them had any abnormality on physical examination and routine blood laboratory tests. No one was taking medication, smoked, or drank alcohol, and all gave informed written consent before participating in this study.

### 2.3. Ferritin, Iron, and Gamma-Glutamyltransferase

Serum ferritin, serum iron (Fe), and gamma-glutamyltransferase (GGT) plasma levels were estimated in healthy controls and MDS patients in the Central National Biochemical Laboratory in the Institute of Hematology and Blood Transfusion. Values of ferritin, iron, and GGT in healthy donors fall within the limits of the reference interval.

### 2.4. Measurement of Total and Reduced Forms of Thiols (Cys, Hcys, GSH, and CG)

Plasma samples were treated according to Raijmakers et al. with several modifications [10]. 60 *μ*L of plasma sample or standards was mixed with 60 *μ*L of PBS and 15 *μ*L of 10% tris(2-carboxyethyl) phosphine (w/v) for total levels of thiols (sum of their reduced and oxidized forms in plasma, including thiols covalently bound to plasma proteins) or with 75 *μ*L of PBS for reduced forms of thiols. Both mixtures were incubated at 25°C for 30 min and deproteinized by the addition of 135 *μ*L of 10% trichloracetic acid with 2 mM EDTA, followed by centrifugation (15000 ×*g*, 15 min, 10°C). To 50 *μ*L of supernatant, 125 *μ*L of 125 mM borate buffer (pH 9.5) with 4 mM EDTA was added, followed by the addition of 15 *μ*L of 1.5 M NaOH and 50 *μ*L of 0.1% ammonium 7-fluorobenzofurazan-4-sulfonate (w/v). The reaction mixture was incubated at 60°C in darkness for 60 min and filtered through 0.2 *μ*m cellulose filters (National Scientific, Rockwood, TN, USA). Chromatographic conditions were used according to Garcia [11], with several modifications using a High Performance Liquid Chromatographic system (HPLC) (Shimadzu, Tokyo, Japan). A 20 *μ*L aliquot was injected into a LUNA C18 (2) column (150 × 3 mm, 5 *μ*m) (Phenomenex, Torrance, CA, USA) and separated at 40°C. The mobile phase was composed of 0.1 M H_3_PO_4_ adjusted to pH 2.1 with KOH (A) and 10% methanol buffer A (B). Elution of the thiol-benzofurazan-4-sulfonate derivatives was performed with a gradient system (*t* (min)/% B: 0/0, 7/0, 7.1/100, 10/100, 10.1/0) at a flow rate of 1 mL/min. The fluorescence signal of excitation was measured at 385 nm and of emission at 515 nm.

### 2.5. Measurement of Nitrite

Nitrite standards and samples were prepared as previously described by Li et al. [12]. Briefly, 200 *μ*L of nitrite standard KNO_2_ (0–625 nM) or a patient sample (10x diluted and ultrafiltered plasma sample) was incubated at 25°C with 20 *μ*L 316 mM 2,3-diaminonaphthalene (in 0.62 M HCl) for 10 min, followed by the addition of 8 *μ*L of triethylamine. The dilution of the sample with water could influence real concentrations of nitrite in plasma with contaminating nitrite in water. This reaction mixture was deproteinized with acetonitrile 1 : 1, centrifuged (17000 ×*g*, 4 min), and filtered through a 0.2 *μ*m cellulose filter (National Scientific). 150 *μ*L of the filtered solution was used directly for the chromatographic separation of reaction product 2,3-naphthotriazole. Chromatographic conditions were used as described by Woitzik et al. [13] with minor changes using a HPLC system (Shimadzu). A 10 *μ*L aliquot of the sample was injected into a Luna C18 (2) column (150 × 3 mm, 5 *μ*m) (Phenomenex). The mobile phase consisted of 30% acetonitrile in 30 mM of phosphate buffer, adjusted to pH 8 with triethylamine. Fluorescence was monitored with excitation at 375 nm and emission at 415 nm, with separations performed at 45°C.

### 2.6. Measurement of Nitrate

Nitrate was determined according to Davies et al. [14] by capillary electrophoresis with UV detection at 214 nm (Beckman Coulter, Fullerton, CA, USA), performed at 25°C with small changes. The components were separated using a −10 kV voltage; reverse electroosmotic flow was used. The separation of samples took place in a fused silica capillary tube (50 *μ*m diameter by 40 cm to the detector), in a buffer consisting of 150 mM NaCl/5 mM Tris-HCl (pH 7.4) and 2 mM tetradecyltrimethylammonium hydroxide (TTAH). TTAH was prepared from a tetradecyltrimethylammonium bromide solution by passing it through a strong anion exchange cartridge (Phenomenex), which replaced the bromide ions with hydroxide ions. The capillary tube was rinsed before each injection with 0.1 M NaOH and a separation buffer for 1 min and 2 min, respectively. Plasma samples were centrifuged (17000 ×g, 4 min, 25°C), filtered through a 0.2 *μ*m cellulose filter (National Scientific), and sonicated. The linearity of the assay was determined by preparing aqueous solutions containing 0.39 *μ*M–500 *μ*M KNO_3_.

### 2.7. Measurement of MDA

Standard and plasma samples were prepared as in our previous study [15]. Briefly MDA standards were prepared by adding 25 *μ*L tetrahydroxypropane into 50 mL of 1% H_2_SO_4_ (v/v). Mixture was incubated in darkness for 2 hours. Concentration of MDA was measured spectrophotometrically (*ε* 245 nm = 13700 cm^−1^· L· mol^−1^). 100 *μ*L of a plasma or standard sample (0–10 *μ*M) was mixed with 12.5 *μ*L of 100 mM EDTA in 2% NaOH (w/v), 12.5 *μ*L of H_2_O or MDA standards, and 125 *μ*L of 10 mM 2,6-ditert-butyl-4-methylphenol in acetonitrile. The mixture was incubated at 60°C for 30 min. Samples were centrifuged (17000 ×*g*, 10 min). To 75 *μ*L of the supernatant, a total of 300 *μ*L of 25 mM 2-thiobarbituric acid in 2 M CH_3_COOH was added (pH 3) and incubated at 100°C for 60 min. Separations were carried out on a 5 *μ*m reversed-phase C18 Gemini NX column (150 × 2 mm) (Phenomenex) at 25°C using a HPLC system (Shimadzu), as was described in our previous work [15]. Elution of the MDA derivative with 2-thiobarbituric acid was performed isocratically with 35% MeOH in 50 mM of NH_4_HCO_3_ buffer, adjusted to pH 9.3 with NH_4_OH at a flow rate of 0.25 mL/min, with UV-Vis detection at 532 nm.

### 2.8. Statistical Analysis

Data are presented as means ± standard deviation (SD) and as a range. A two-tail-, -two sample Student's *t*-test was used to compare MDS patients with healthy donors. One-way ANOVA was computed to examine the differences across all groups (MDS, healthy controls). Post hoc analyses using Duncan homogeneous subsets were performed for the cases in which the main effect was significant. A Pearson correlation test was used for the normally distributed data and a Spearman's rank correlation test for nonparametric data. All tests for statistical significance were standardized at an alpha level of *P* < 0.05.

All methods used were performed according to standard operating procedures (SOP) validated and verified. The methods have been optimized and validated for selectivity, precision, and recovery using an internal quality control. All of the tested compounds analyzed by chromatography methods had linearity of >98%, with relative standard deviation <10% in terms of variation of retention time. Interday and intraday variability was <5%.

## 3. Results

### 3.1. Oxidative Stress Parameters in MDS Patients and Healthy Donors

Plasma levels of total, oxidized, and reduced forms of aminothiols Cys, Hcys, and CG were elevated in the plasma of all MDS patients, when compared with healthy controls using a two-tailed, two-sample Student's *t*-test. Conversely, plasma levels of total (t-GSH) and oxidized (ox-GSH) forms of GSH and nitrite were significantly depressed in all MDS patients, comparing with healthy donors. We did not observe any significant differences between MDS patients and healthy donors in plasma levels of MDA and nitrate. Serum ferritin levels exceeded the upper limit of the reference interval in MDS patients. The means of free iron serum levels were in the reference interval and levels of GGT were at the upper limit edge of the reference interval in all MDS patients. Data are shown in Tables 1 and 2.

### 3.2. Oxidative Stress Parameters in MDS Patients in the Context of Clinical Outcomes

**Figure 1:**
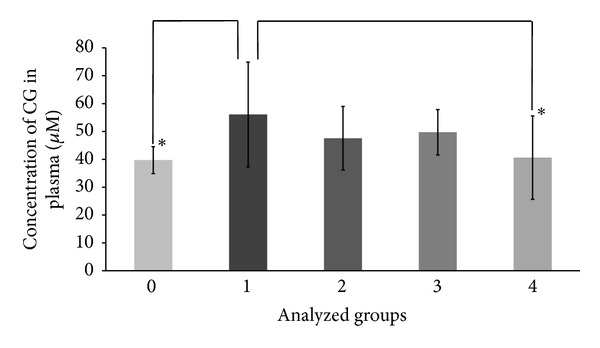
Concentration of total cysteinylglycine (t-CG). Plasma levels of t-CG (reduced form and form bound to proteins) in healthy donors (0) and MDS subgroups (1–4). Data are expressed as means ± SD. Using ANOVA, t-CG was found to differ significantly between groups (*P* = 0.032). *Statistical significance of the difference between group 1 and both groups 0 and 4 (post hoc ANOVA tests, Duncan, *P* < 0.05).

**Figure 2:**
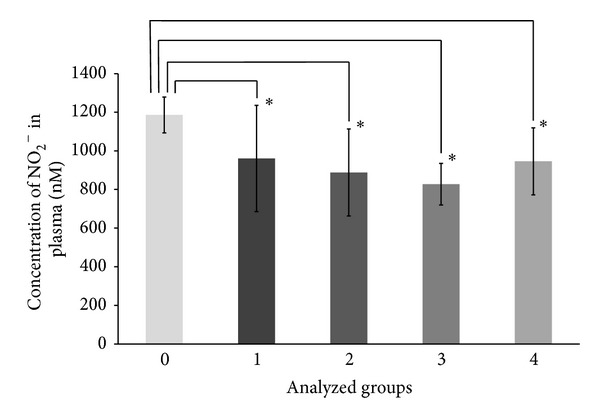
Concentration of nitrite (NO_2_^−^). Plasma levels of nitrite in healthy donors (0) and MDS subgroups (1–4). Data are expressed as means ± SD. Using ANOVA, nitrite was found to differ significantly between groups (*P* = 0.021). *Statistical significance of the difference between groups 1–4 and the control group (post hoc ANOVA tests, Duncan, *P* < 0.05).

Subsequently, MDS patients were divided into four study groups (1–4) according to their common clinical and diagnostic outcomes (Table 3). The groups were compared with each other and with a group of healthy donors (0). Table 3 provides an overview of the groups analyzed in the presented study. ANOVA was used to test for significant differences in the means of measured compounds concentrations between the analyzed groups. As shown in Table 4, significant differences were observed between the analyzed groups for total CG (t-CG) (*P* = 0.032) and for nitrite (*P* = 0.021). Oxidized CG (ox-CG) also significantly differed between groups (*P* = 0.029). Using post hoc ANOVA tests (Duncan) we ascertained that the levels of t-CG (Figure 1) and ox-CG were significantly higher in group 1 of MDS patients, with respect to the healthy donors and group 4. The same post hoc test showed the levels of nitrite significantly lower in all MDS subgroups as compared to the healthy controls (Figure 2).

Plasma levels of all forms of Cys were also higher in all MDS subgroups, as compared to the healthy donors. The highest plasma levels were found mostly in groups 1 and 2, respectively. Total, oxidized, and reduced GSH concentrations tended to be lower in all MDS patients, as compared to the healthy donors; however, there was not a statistical significance between the groups. The levels of MDA were higher in groups 1 and 3, as compared with healthy donors; but these data differences were also not statistically significant. Plasma levels of nitrate in groups 0, 1, and 2 were approximately the same; lower values were observed in groups 3 and 4, with the lowest value in group 4; yet they still did not differ significantly. Levels of serum free iron and serum ferritin did not differ significantly between the MDS groups.

**Figure 3:**
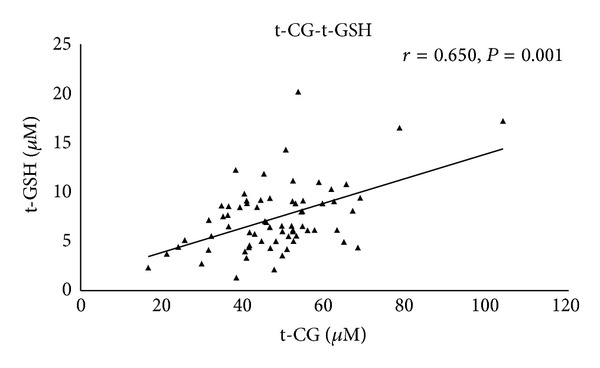
Correlation of total cysteinylglycine (t-CG) and total glutathione (t-GSH). Correlation of t-CG plasma concentrations and t-GSH plasma concentrations in MDS patients and healthy donors. *P* and *r* values were derived by a Pearson correlation test.

**Figure 4:**
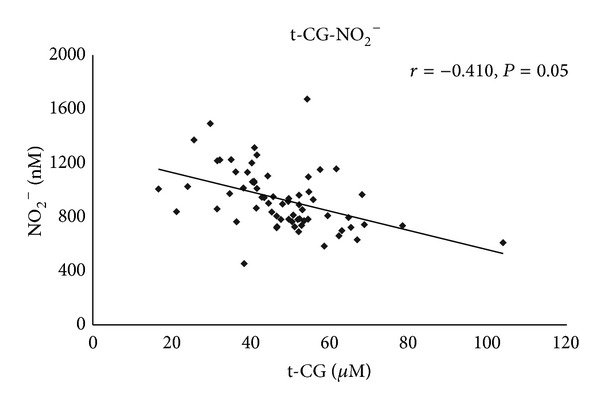
Correlation of cysteinylglycine (t-CG) and nitrite (NO_2_^−^). Correlation of t-CG plasma concentrations and nitrite plasma concentrations in MDS patients and healthy donors. *P* and *r* values were derived by a Pearson correlation test.

Using a Pearson correlation test, a moderate positive correlation was found between all forms of evaluated thiols. Moreover, reduced Cys (red-Cys) correlated moderately with t-CG (*r* = 0.40, *P* = 0.001) and moderately with reduced CG (red-CG) (*r* = 0.77, *P* = 0.001). A moderate correlation between the levels of t-CG and t-GSH (*r* = 0.65, *P* = 0.001) (Figure 3) and moderate correlation between both ox-CG (*r* = 0.48, *P* = 0.001) and red-CG (*r* = 0.39, *P* = 0.001) and t-GSH was found. Moreover, a moderate negative correlation was found between the concentrations of all forms of CG: t-CG (*r* = −0.41, *P* = 0.005), ox-CG (*r* = −0.40, *P* = 0.001), and red-CG (*r* = −0.49, *P* = 0.001), and nitrite (Figure 4).

### 3.3. Oxidative Stress Parameters in MDS Patients in the Context of Iron Overload

Patients were divided according to the possible risk of iron overload (high iron and ferritin levels) into a group of patients requiring chelation therapy (CH) (16) and a group of patients not requiring chelation therapy (non-CH) (30). Applying a two-tailed, two-sample Student's *t*-test, we estimated that the levels of serum free iron (*P* = 0.001) and serum ferritin (*P* = 0.006) were significantly higher in the CH group. However, no significant differences in aminothiol, nitrite, and nitrate levels were found between these two groups. MDA concentrations were significantly higher in the group CH (*P* = 0.001). Furthermore, we compared analyzed compounds in patients regularly receiving blood transfusions (T) with patients who were not receiving blood transfusions (non-T). We found significantly higher levels of serum free iron (*P* = 0.004) and MDA (*P* = 0.013) in the group T of patients. No significant differences were found between the groups for the other analyzed compounds.

**Figure 5:**
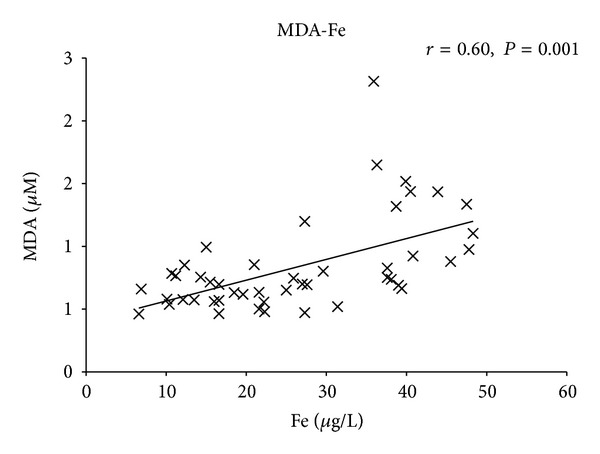
Correlation of total malondialdehyde (MDA) and free iron (Fe). Correlation of MDA plasma concentrations and Fe serum concentrations in MDS patients and healthy donors. *P* and *r* values were derived by a Pearson correlation test.

**Figure 6:**
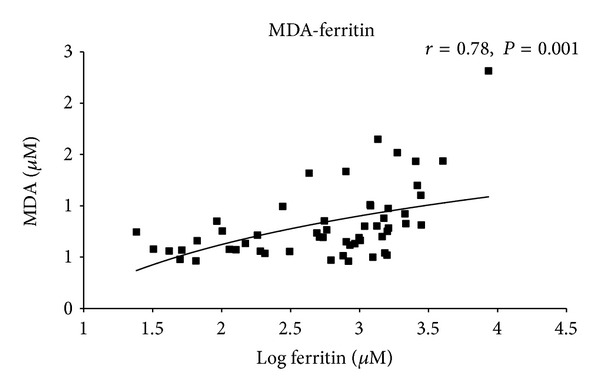
Correlation of total malondialdehyde(MDA) and ferritin. Correlation of MDA plasma concentrations and serum ferritin concentrations in MDS patients and healthy donors. Values of ferritin are in decadic logarithm and regression is logarithmic. *P* and *r* values were derived by a *Pearson* correlation test.

Our data showed that while a lack of correlation was found between plasma oxidative stress parameters (aminothiols, nitrite, and nitrate) and serum iron levels/ferritin levels, MDA correlated moderately with both serum free iron levels (*r* = 0.60, *P* = 0.001) (Figure 5) and serum ferritin levels (*r* = 0.78, *P* = 0.001) (Figure 6).

## 4. Discussion

Our results revealed that plasma concentrations of total, oxidized, and reduced forms of Cys, Hcys, and CG were significantly elevated in MDS patients versus healthy donors; conversely, plasma levels of total and oxidized GSH and nitrite were significantly depressed in MDS patients compared to the control group. Moreover, significant concentration differences of nitrite, t-CG, and ox-CG were found between the clinical subgroups of MDS patients and the controls. We also found that patients requiring chelation therapy and those receiving transfusions had significantly higher levels of both MDA and free iron, whereas aminothiols, nitrite, and nitrate compounds did not differ between these groups and the compared patient groups not requiring chelation treatment or the nontransfused patients. The question arises of what is the cause and significance of these newly elucidated findings.

We found significantly depressed levels of nitrite in all groups of MDS patients versus controls. These results were in accordance with our previous study of middle age patients with MDS [16]. However, the age of the subjects in our study group, was in the range 25–91 years. The relation of plasma levels of nitric oxide to the patient age depends on the substances (NO_2_^−^, NO_3_^−^) measured to assess NO [17–19]. Alusik et al. found that nitrite levels in elderly patients (over eighty) were slightly, nonsignificantly lower than in a younger control group in their thirties, whereas nitrate concentrations were nonsignificantly higher in elderly patients than in these controls [20]. Mikiwa et al. found that together, both males and females showed a nonsignificant enhancement of nitrite and nitrate with age [21]. Even the levels of aminothiols are believed to vary with age. Bates et al. found in a study of young people aged 4–18 years, compared with people aged 65 years and over, that both Hcys and Cys exhibited progressive increases with age throughout the age range whereas CG plasma concentration did not change significantly with age [22]. According to our previous discussions a correct assessment of the importance of nitric oxide and CG levels estimated in our MDS patients should not be influenced by age, because these relations were referred to as non-significant, as shown above. Collecting a control group of healthy individuals of older age is rather problematic, due to other diseases typical for the elderly population (hypertension, diabetes, etc.). Optimally, the control group should match for as many parameters as possible. As MDS usually occurs in elderly patients, the control group should be of similar age range. However, this matching may face other limitations—the primary difficulty is to find healthy individuals of older age who do not suffer from other abovementioned diseases which may significantly affect the obtained results. Further, MDS is not limited to elderly patients only. Considering that, we decided to compare our patient group with a healthy control group of lower age range to possibly observe all the changes that would occur.

**Figure 7:**
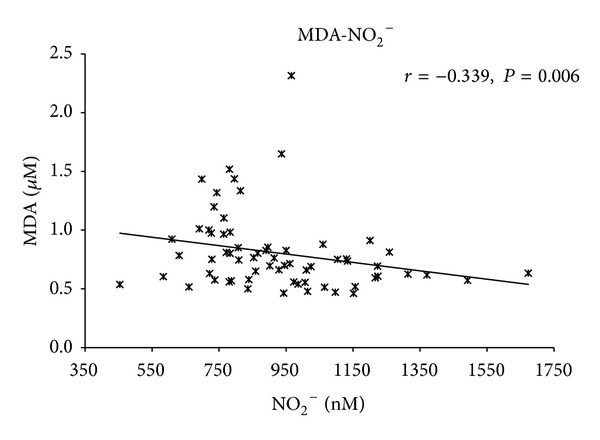
Correlation of total malondialdehyde (MDA) and nitrite (NO_2_^−^). Correlation of MDA plasma concentrations and nitrite plasma concentrations in MDS patients and healthy donors. *P* and *r* values were derived by a Spearman's rank correlation test.

Association between decreased nitrite concentration and oxidative stress (increased levels of MDA) in healthy humans has been described [23]. Modun et al. found significantly decreased levels of plasma nitrite and significantly enhanced levels of MDA in healthy donors after hyperoxia. The authors supposed the role of the generated ROS and their rapid reaction with NO in generating peroxynitrite and hence decrease in NO bioavailability. They also considered diminish production of NO by endothelial NO synthase (eNOS) due to the decrease of cofactor of eNOS, tetrahydrobiopterin, through its oxidation or eNOS uncoupling in the presence of ROS and peroxynitrite. In our study the significantly lowered levels of nitrite in the studied group of MDS patients versus the controls could be induced by a combination of more than one factor. One consideration is the possible consumption of NO by an elevation of plasma free hemoglobin levels [24]. Thus, we further sorted the patients into transfused and nontransfused groups. Consequently, we tested the hypothesis that transfusion lowered patient nitrite levels, as a consequence of NO consumption by free hemoglobin. In the samples of patients who received transfusions, we did not find significantly lowered levels of nitrite compared to patients without transfusions (*P* = 0.137; two-tailed, two-sample Student's *t*-test), although they tended to be lower. We found significantly enhanced levels of MDA in transfused patients. Moreover, nitrite concentrations moderately negatively correlated with MDA levels (*r* = −0.339, *P* = 0.006) (Figure 7), using a Spearman's rank correlation test. It has been described that free hemoglobin in plasma may increase MDA concentrations in plasma [25] and hence could contribute to oxidative stress in MDS patients. Enhanced oxidative stress may oxidize tetrahydrobiopterin, the crucial cofactor of eNOS, and its deficiency may result in eNOS uncoupling [26]. Furthermore, eNOS could be inhibited by enhanced levels of asymmetric dimethylarginine which has been described in plasma of MDS patients [16]. Our study does not reveal the underlying mechanism of depressed concentration in nitrite in plasma of MDS patients. We suppose that oxidative stress in MDS patients could contribute to disruption of eNOS. However, differences in age, diet, and consumption by free hemoglobin should be also considered. This problem remains to be elucidated in further studies.

A negative correlation of plasma nitrite with all forms of CG was found in our study. We also found significantly higher plasma levels of CG together with Cys and Hcys. Aminothiols, especially Hcys, are a common cardiovascular risk factor. In our study there were 19 patients with cardiovascular event and 28 patients with hypertension. We tested hypotheses that patients with cardiovascular event or hypertension had higher levels of Cys, CG, and Hcys. Using two-tailed, two-sample Student's *t*-test we have found significant differences neither in Hcys levels nor in levels of CG among groups of patients with cardiovascular event and hypertension comparing to group of patients without mentioned comorbidities. Only levels of total Cys were significantly higher in patients with hypertension (*P* < 0.03). The higher levels of total Hcys in MDS have been described earlier and were in accordance with Cortelezzi et al. [6]. Elevated levels of Cys had not yet been described in relation with MDS. De Chiara et al. studied Cys in cardiovascular disease, and they have suggested that Cys is the main plasma antioxidant compound, with its concentration reflecting increased oxidative processes [27]. However, an association has been described between Cys and older age [28]. As mentioned, CG was described to have a lack in correlation with age [22]. This dipeptide has not been as widely studied as GSH and Hcys; however, its role in the pathophysiology of several diseases has been described previously [29]. We found that levels of all forms of CG were significantly higher in MDS patients, and moreover, levels of t-CG differed significantly between each of the MDS clinical subgroups and levels of ox-CG were significantly higher in subgroup 1 of MDS compared to subgroup 4 and healthy donors. CG together with Cys is the main components of GSH metabolism and essential substrates for GSH synthesis.

Reduced levels of plasma GSH as a consequence of oxidative stress have been described in several works [30–32]. Intracellular GSH is a key antioxidant involved in the protection of the cell against oxidative radicals forming GSH disulfide and in the metabolism of endogenous and xenobiotic compounds to yield mostly thioethers. GSH has also been described to play a critical role in determining apoptosis sensitivity and resistance in leukemia cells [33, 34]. Once GSH is oxidized, it is exported out of the cell and degraded [35]. GSH is catabolized through the action of GGT to *γ*-glutamyl moiety, coupled to another amino acid and CG, which can be further catabolised to Cys and glycine. Significantly reduced levels of total and oxidized GSH, together with enhanced levels of Cys and CG, suggest a possible imbalance in GSH metabolism in MDS. In our study, the activity of GGT, a crucial enzyme in the metabolism of GSH in MDS patients, was 0.60 *μ*kat/L, which is mostly at the upper limit of the standard reference interval of GGT (0.16–0.8 *μ*kat/L). Moreover, levels of GGT were moderately negatively correlated with total GSH concentrations. GGT has been described as a marker of oxidative stress [31–33], and several studies support the view that the enhanced expression of GGT may represent an important factor in the development of a more aggressive and resistant phenotype of cancer cells [36–38]. In addition, De Donatis et al. showed that the blocking of GSH metabolism through GGT inhibition elicited an extralenticular accumulation of GSH and the ability of CG to abolish this effect [39]. In our study, the significantly elevated levels of CG as a consequence of enhanced GGT activity could be considered.

Ghoti et al. described lower levels of reduced GSH in the red blood cells, platelets, and neutrophils of MDS patients with RARS and RCMD. These data correlated with serum ferritin levels and were attributed to oxidative stress due to iron overload [4]. In our study, MDS patients had significantly depressed plasma levels of t-GSH and ox-GSH as compared with healthy donors. In several works, oxidative stress in MDS was attributed to the early stages of MDS, characterized by enhanced apoptosis and transfusion therapy [2, 40]. These patients suffer from iron overload and consequently oxidative stress development [41]. We found enhanced plasma levels of aminothiols (Cys, CG, and Hcys) in groups 1 and 2, respectively. These groups involve MDS subtypes designated as early stages of MDS. However, serum ferritin levels and serum free iron did not differ significantly between each of the MDS subgroups (1–4) in our study. Meanwhile, the oxidative stress represented by MDA concentrations was significantly higher in patients with iron overloads (meaning patients requiring chelation therapy and transfused patients). MDA had a moderate correlation with iron and ferritin levels. However, other analyzed compounds lacked correlation with oxidative stress as a consequence of iron overload. This conflict with Ghoti et al. may be explained by experimental conditions. We evaluated levels of GSH in plasma and Ghoti et al. did so in cells. We suggest possibility that there is an oxidative stress as a result of iron overload; however, the imbalance in plasma aminothiols, nitrite, and nitrate compound could be probably influenced by other factors.

## 5. Conclusions

In conclusion, we simultaneously determined nitrite, and nitrate; plasma aminothiols, and MDA in 61 MDS patients in the context of clinical outcomes and as a consequence of iron overload and compared both with 23 healthy donors. Our results revealed for the first time the significant differences in the concentrations of total plasma aminothiols in MDS patients and no correlation of these parameters with iron overload represented by serum iron/ferritin levels. We suggest that oxidative stress could participate in the development of MDS disease, not only to be consequence of iron overload. This work brings about new insight into the problematic nature of MDS and oxidative stress. However, further studies are needed to clarify this subject more concretely.

## Figures and Tables

**Table 1:** Plasma levels of oxidative stress compounds in healthy donors and myelodysplastic patients (MDS). Data are expressed as means ± SD. Significant differences between MDS patients and healthy donors are marked with ∗. Data were analyzed using a two-tailed, two-sample Student's *t*-test.

Analyzed compounds	Healthy donors (*n* = 23)	MDS patients (*n* = 61)
t-Cys (µM)	219 ± 14	284 ± 68***
t-Hcys (µM)	9.45 ± 0.63	14.50 ± 10.70***
t-CG (µM)	39.71 ± 4.86	48.80 ± 14.30**
t-GSH (µM)	9.07 ± 1.55	7.16 ± 3.59*
MDA (µM)	0.69 ± 0.11	0.82 ± 0.34
NO_2_^−^ (nM)	1149 ± 86	903 ± 215***
NO_3_^−^ (µM)	32.78 ± 10.33	32.80 ± 17.87
Ferritin (µg/L)	Ref. R.: 22–322	557.9 ± 4.0
Fe (µM)	Ref. R.: 7.2–29	26.15 ± 12.28
GGT (µkat/L)	Ref. R.: 0.14–0.8	0.60 ± 0.56

***P < 0.001, **P < 0.005, *P < 0.05, total cysteine (t-Cys), total homocysteine (t-Hcys), total cysteinylglycine (t-CG), total glutathione (t-GSH), malondialdehyde (MDA), and gamma-glutamyltransferase (GGT).

**Table 2:** Plasma levels of total, reduced, and oxidized forms of thiols in healthy donors and myelodysplastic patients (MDS). Data are expressed as means ± SD. Significant differences between MDS patients and healthy donors are marked with ∗. Data were analyzed using a two-tailed, two-sample Student's *t*-test.

Analyzed compounds	Healthy donors (*n* = 23)	MDS patients (*n* = 61)
t-Cys (µM)	219 ± 14	284 ± 68***
red-Cys (µM)	4.06 ± 1.07	8.10 ± 3.46***
ox-Cys (µM)	215 ± 14	275 ± 67***
t-Hcys (µM)	9.45 ± 0.63	14.50 ± 10.70***
red-Hcys (µM)	0.03 ± 0.01	0.05 ± 0.07*
ox-Hcys (µM)	9.43 ± 0.63	14.45 ± 10.64***
t-CG (µM)	39.71 ± 4.86	48.8 ± 14.30**
red-CG (µM)	1.50 ± 0.51	3.35 ± 2.33***
ox-CG (µM)	38.21 ± 4.57	45.44 ± 12.72*
t-GSH (µM)	9.07 ± 1.55	7.16 ± 3.59*
red-GSH (µM)	0.39 ± 0.09	0.41 ± 0.39
ox-GSH (µM)	8.68 ± 1.48	6.74 ± 3.26*

***P < 0.001, **P < 0.005, *P < 0.05, total cysteine (t-Cys), reduced cysteine (red-Cys), oxidised cysteine (ox-Cys), total homocysteine (t-Hcys), reduced homocysteine (red-Hcys), oxidised homocysteine (ox-Hcys), total cysteinylglycine (t-CG), reduced cysteinylglycine (red-CG), oxidised cysteinylglycine (ox-CG), total glutathione (t-GSH), reduced glutathione (red-GSH), and oxidised glutathione (ox-GSH).

**Table 3:** Overview of the analyzed study groups (0–4), the number of myelodysplastic patients, gender ratio, and the age of analyzed patients and controls.

Group	Number of patients (male/female)	Age range	Diagnoses
0	23 (10/13)	25–57	Healthy donors
1	14 (10/4)	52–91	RA, RA-5q, RARS
2	29 (15/14)	28–90	RCMD, RCMD-RS, RCMD-5q, RCMD with reactive monocytes
3	9 (4/5)	36–85	RAEB-1, MDS/MPS, MDS/MPS with 5q deletion, MDS-RAEB-1 + SC-NHL
4	9 (5/4)	55–80	RAEB-2, RAEB-T, AML M2

Refractory anemia (RA), MDS with isolated del(5q) (RA-5q), refractory anemia with ringed sideroblasts (RARS), refractory cytopenia with multilineage dysplasia (RCMD), RCMD with ringed sideroblasts (RCMD-RS), RCMD with 5q deletion (RCMD-5q), RCMD with reactive monocytes, RA with an excess of blasts-1 (RAEB-1), RA with an excess of blasts-2 (RAEB-2), RA with an excess of blasts in transformation (RAEB-T), myelodysplastic/myeloproliferative syndromes (MDS/MPS), MDS/MPS with 5q deletion, acute myeloid leukemia (AML M2), and MDS-RAEB-1 plus SC-non-Hodgkin lymphoma.

**Table 4:** Oxidative stress parameters in the control group (0) and four myelodysplastic syndromes subgroups (1–4). Values are shown as means ± SD. Data were analyzed by ANOVA, and *P* values are shown for significantly differing parameters.

Analyzed compounds	Analyzed groups					*P* value
	0	1	2	3	4	
t-Cys (µM)	219 ± 14	300 ± 57	284 ± 71	274 ± 56	268 ± 90	
t-Hcys (µM)	9.45 ± 0.63	11.73 ± 4.87	17.12 ± 14.51	13.50 ± 3.17	11.38 ± 4.75	
t-CG (µM)	39.71 ± 4.86	56.09 ± 18.81	47.54 ± 11.41	49.70 ± 8.15	40.59 ± 14.95	*0.032
t-GSH (µM)	9.07 ± 1.55	8.36 ± 4.27	6.83 ± 2.88	7.25 ± 4.89	6.26 ± 3.22	
MDA (µM)	0.70 ± 0.12	0.99 ± 0.51	0.74 ± 0.25	0.92 ± 0.31	0.71 ± 0.15	
NO_2_^−^ (nM)	1185 ± 93	960 ± 275	890 ± 225	827 ± 108	945 ± 173	*0.021
NO_3_^−^ (µM)	34.80 ± 11.16	33.14 ± 13.87	37.47 ± 20.32	29.29 ± 18.91	20.78 ± 6.91	
Ferritin (µg/L)	Ref. R.: 22–322	1569 ± 2	330 ± 6	1681 ± 2	250 ± 3	
Fe (µM)	Ref. R.: 7.2–29	38.19 ± 9.13	25.23 ± 13.15	29.44 ± 7.20	19.51 ± 10.1	
GGT (µkat/L)	Ref. R.: 0.14–0.8	0.75 ± 0.77	0.70 ± 0.71	0.60 ± 0.17	0.42 ± 0.27	

Total cysteine (t-Cys), total homocysteine (t-Hcys), total cysteinylglycine (t-CG), total glutathione (t-GSH), malondialdehyde (MDA), and gamma-glutamyltransferase (GGT).
